# Elastic Self-Recovering Hybrid Nanogenerator for Water Wave Energy Harvesting and Marine Environmental Monitoring

**DOI:** 10.3390/s24123770

**Published:** 2024-06-10

**Authors:** Qiuxiang Wang, Gao Yu, Ying Lou, Mengfan Li, Jiaxi Hu, Jiaodi Li, Weiqi Cui, Aifang Yu, Junyi Zhai

**Affiliations:** 1 Center on Nanoenergy Research, Institute of Science and Technology for Carbon Peak & Neutrality, Key Laboratory of Blue Energy and Systems Integration (Guangxi University), Education Department of Guangxi Zhuang Autonomous Region, School of Physical Science & Technology, Guangxi University, Nanning 530004, China; 2 Beijing Key Laboratory of Micro-Nano Energy and Sensor, Center for High-Entropy Energy and Systems, Beijing Institute of Nanoenergy and Nanosystems, Chinese Academy of Sciences, Beijing 101400, China; 3 School of Nanoscience and Engineering, University of Chinese Academy of Sciences, Beijing 100049, China

**Keywords:** hybrid nanogenerator, elastic self-recovering, water wave energy harvesting, marine environmental monitoring

## Abstract

To achieve large-scale development of triboelectric nanogenerators (TENGs) for water wave energy harvesting and powering the colossal sensors widely distributed in the ocean, facile and scalable TENGs with high output are urgently required. Here, an elastic self-recovering hybrid nanogenerator (ES-HNG) is proposed for water wave energy harvesting and marine environmental monitoring. The elastic skeletal support of the ES-HNG is manufactured using three-dimensional (3D) printing technology, which is more conducive to the large-scale integration of the ES-HNG. Moreover, the combination of a TENG and an electromagnetic generator (EMG) optimizes the utilization of device space, leading to enhanced energy harvesting efficiency. Experimental results demonstrate that the TENG achieves a peak power output of 42.68 mW, and the EMG reaches a peak power output of 4.40 mW. Furthermore, various marine environment monitoring sensors, such as a self-powered wireless meteorological monitoring system, a wireless alarm system, and a water quality monitoring pen, have been successfully powered by the sophisticated ES-HNG. This work introduces an ES-HNG for water wave energy harvesting, which demonstrates potential in marine environment monitoring and offers a new solution for the sustainable development of the marine internet of things.

## 1. Introduction

The ocean, covering 71% of the Earth’s surface, harbors abundant renewable resources essential for human survival [1,2]. However, the dynamic environment and complex climate of the ocean present significant safety hazards, so monitoring of the marine environment is of paramount importance [3,4,5]. Real-time monitoring and data collection in the marine environment are facilitated by self-powered marine sensors, which have become indispensable tools [6,7,8,9]. This provides important information support for our ocean protection and management. Currently, sensor power in the ocean is primarily dependent on batteries. However, the environmental pollution and ecosystem disruption caused by battery usage, as well as the significant investment in human and material resources for battery maintenance, increase the cost and complexity of ocean monitoring [10,11,12,13,14]. Therefore, there is an urgent requirement for a clean, efficient, and cost-effective energy harvesting method to power sensors in the ocean.

The triboelectric nanogenerator (TENG) is an emerging energy harvesting technology pioneered by Wang’s team in 2012. Its working principle is based on the coupling of triboelectrification and the electrostatic induction effect [15,16]. This technology offers various advantages such as being lightweight, cost-effective, and offering a diverse choice of materials [17,18,19,20,21]; hence, it has received widespread attention and research. In particular, the TENG demonstrates high efficacy in capturing wave energy at low frequencies, making it a viable solution for harvesting ocean wave energy and providing a dependable energy supply for numerous sensors distributed in the ocean [22,23,24,25]. Numerous studies have investigated the utilization of TENGs for harvesting water wave energy, including pendulum-type structures [26,27,28] and cylindrical structures [29,30,31]. However, the movement mechanisms and transmission structures of these designs require a large amount of space, thus leading to low space utilization. Additionally, designs such as gear structures [32,33,34] and spring structures [35,36,37] have complex manufacturing processes and result in mechanical energy losses during energy conversion. Moreover, several studies have integrated a TENG and an electromagnetic generator (EMG) to achieve dual-form energy collection, enhancing spatial utilization and energy capture efficiency [38,39,40,41]. It can be seen that to achieve efficient water wave energy harvesting, TENGs need to be designed with simple structures that are easy to manufacture on a large scale. During the energy conversion process, energy loss should be avoided. Combining the TENG and the EMG is an efficient method that can improve the spatial utilization of the device.

In this study, an elastic self-recovering hybrid nanogenerator (ES-HNG) is proposed to achieve water wave energy harvesting and environmental monitoring. The elastic skeleton of ES-HNG is manufactured using three-dimensional (3D) printing, which facilitates its large-scale production. ES-HNG is composed of both a TENG module and an EMG module, fully utilizing the space of the device. Through the optimization of internal structural parameters and external influencing factors, the TENG achieves a peak power of 42.68 mW, while the EMG achieves a peak power of 4.40 mW. Subsequently, the output performance of the ES-HNG under different water wave conditions is explored, with the TENG achieving a peak power of 7.44 mW and the EMG achieving a peak power of 0.10 mW. Finally, by integrating the ES-HNG with a suitable power management circuit (PMC), this successfully provides a reliable energy supply for a self-powered wireless meteorological monitoring system, a wireless alarm, and a water quality monitoring pen.

## 2. Materials and Methods

### 2.1. Manufacturing of ES-HNG

Manufacturing of the TENG: The elastic skeleton (bottom 9 cm × 9 cm, height 5 cm) was designed using SolidWorks software (SolidWorks 2021) and printed with a 3D printer (Raise3D, Shanghai, China) using thermoplastic polyurethane (TPU) material. Each elastic skeleton could accommodate six pairs of frictional layer power generation modules. The method for making one pair of frictional layer modules was as follows: the size of each material was 3.5 cm × 9 cm, with copper film used as the electrode, polyamide (PA) as the positive electrode material, fluorinated ethylene propylene (FEP) as the negative electrode material, and a sponge placed under the copper film was used as a base buffer material to ensure sufficient contact of the friction layer. We used high-voltage polarization to pretreat the surface of the friction layer. Manufacturing of the EMG: An 8 cm × 8 cm × 5 mm magnet was placed on the top of the elastic skeleton as a counterweight. At the bottom of the structure, a coil with an outer diameter of 70 mm, an inner diameter of 4 mm, a bottom thickness of 5 mm, and a wire diameter of 0.05 mm was placed.

### 2.2. Characterization and Measurement

The motion of the ES-HNG was simulated using a linear motor (LinMot: BF01-37, Spreitenbach, Switzerland). The short-circuit current and transferred charge of the ES-HNG were measured using a programmable electrometer (Keithley 6514, Beaverton, OR, USA). The open-circuit voltage was measured using a digital oscilloscope (LeCroy 610Zi, New York, NY, USA).

## 3. Results and Discussion

**Figure 1:**
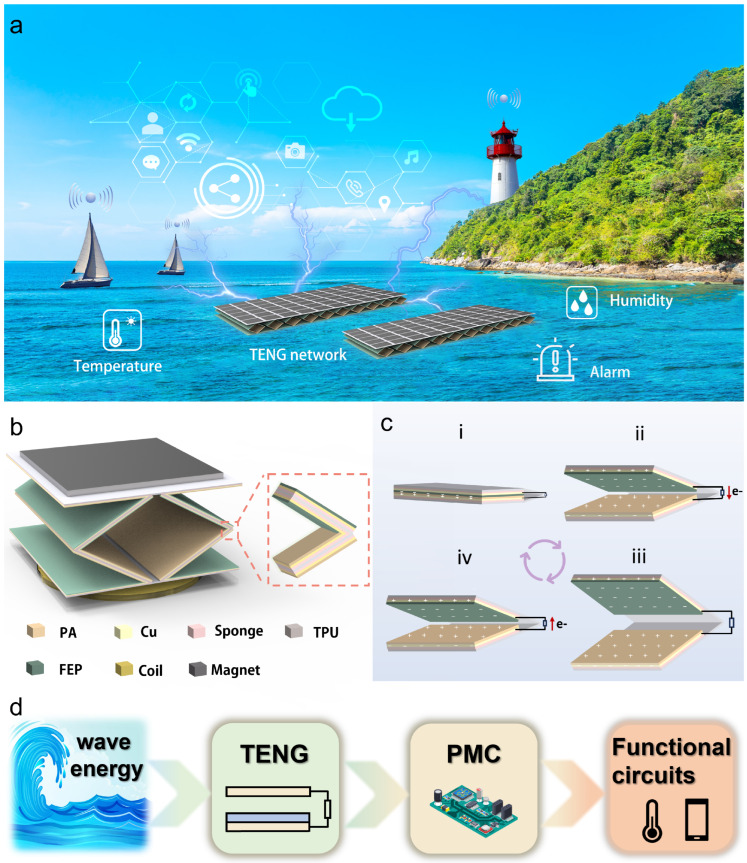
Structure and working principle of ES-HNG: (**a**) the application scenario of the ES-HNG network in the marine internet of things; (**b**) structural schematic diagram and partial magnified view of the ES-HNG device; (**c**) working principle diagram of the TENG; (**d**) process schematic of the TENG for self-powered environmental monitoring.

The application scenario of the ES-HNG network in the marine internet of things is shown in Figure 1a. Figure 1b presents the structural schematic diagram of a single ES-HNG, and its physical diagram is shown in Figure S1a. The ES-HNG is composed of a combination of a TENG and an EMG. The TENG utilizes a TPU elastic skeleton for support, with the schematic of the elastic skeleton shown in Figure S1b. A PA film is the positive friction layer, a FEP film is the negative friction layer, a copper film is the electrode, and a sponge serves as the base buffer material to enhance the contact of the friction layer. The EMG is composed of a copper coil placed at the bottom of the TPU elastic skeleton and a magnet placed at the top as a counterweight. The working principle of ES-HNG under the combined action is shown in Figure S2. Under the driving force of water waves, the magnet at the top operates with the elasticity of the structure, allowing the friction layer of the TENG to contact and separate. At the same time, the magnetic flux in the EMG coil changes, thereby generating current in the coil. The working principle of the TENG is illustrated in Figure 1c. At the initial stage, the friction layers of the structure are in contact, during which the PA film attains a positive charge and the FEP film attains a negative charge, as shown in Figure 1c(i). As the magnet ascends, the friction layer initiates separation, prompting electrons to flow from the FEP film electrode to the PA film electrode through the external load, as shown in Figure 1c(ii). When the friction layer is completely separated, it reaches the electrostatic equilibrium state, as shown in Figure 1c(iii). As the friction layer approaches again, electrons flow from the PA film electrode to the FEP film electrode through the external load, as shown in Figure 1c(iv). For the practical application of ES-HNG in the ocean, it is necessary to construct a self-powered energy harvesting and utilization system with high efficiency, as shown in Figure 1d. The motion of water waves drives the ES-HNG, thereby converting the mechanical energy of the water waves into electrical energy. Through PMC, electrical energy can be stored in capacitors to power electrical devices. Additionally, ES-HNG achieves a peak power density of 105.68 W/m^3^. Figure S3 compares the output performance of the ES-HNG with the previous classic works. Compared with some previous similar works, ES-HNG has superior output performance.

**Figure 2:**
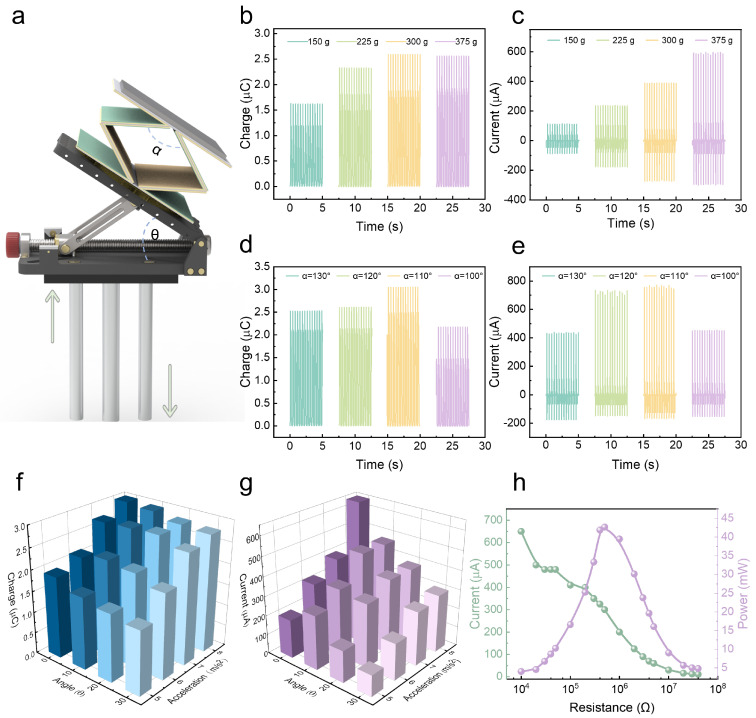
The output performance of the TENG under different parameters: (**a**) schematic diagram of the motor and tilt angle platform; the effect of the counterweight change on (**b**) output transferred charge and (**c**) output short-circuit current; the effect of the elastic skeletal angle α change on (**d**) output transferred charge and (**e**) output short-circuit current; the effect of acceleration and tilt angle θ on the (**f**) output transferred charge and (**g**) output short-circuit current; (**h**) the output current and power-resistance change curve of the TENG.

To investigate the output performance of the ES-HNG, a vertically placed linear motor is used to drive the ES-HNG, and an angle adjustment platform is used to simulate the impact of the tilt angle of the ES-HNG under wave conditions on the output performance. The schematic diagram of the linear motor and the tilt angle platform is shown in Figure 2a. Various factors, such as magnet weight, the angle α of the elastic skeleton, tilt platform angle θ, and motor acceleration, may have a significant impact on the output performance of the TENG. Hence, a comprehensive exploration of these influencing factors is deemed necessary. The weight of the magnet plays a crucial role in determining the movement state and output performance of the TENG, so the effect of the weight change of the magnet on the output performance of the TENG is first investigated. The results in Figure 2b,c indicate that as the weight of the magnet increases from 150 g to 375 g, the transferred charge increases from 1.63 μC to 2.60 μC, the short-circuit current increases from 113.81 μA to 592.68 μA, and the open-circuit voltage increases from 703.83 V to 1327.87 V (Figure S4). This is due to the increased weight of the magnet, which allows the friction layer of the TENG to make more comprehensive contact. However, excessive weight may lead to over-stacking between the friction layers, impeding their swift separation. Based on this, a magnet with a weight of 375 g is selected for subsequent testing. The angle α of the elastic skeleton structure significantly impacts the overall height of the ES-HNG, which may affect the output performance of the TENG. Hence, it is essential to investigate how the angle α of the elastic skeleton influences the output performance of the TENG. The results demonstrate that as the angle α gradually decreases from 130° to 100°, the output performance in terms of transferred charge and short-circuit current initially increases and then decreases. When α is 110°, the optimal transferred charge is 3.04 μC, and the short-circuit current is 760 μA, as shown in Figure 2d,e. The change in the angle α of the elastic skeleton and height follows the equation:

(1) $$L\cos\frac{\alpha }{2}=\frac{H}{2},$$

where *L*, *α*, and *H* represent the side length, angle, and height of the elastic skeleton, respectively. A diagram of the relationship between the angle *α* and height *H* of the ES-HNG elastic skeleton is shown in Figure S5. When *α* is excessively large, the overall height *H* of the elastic skeleton decreases, leading to reduced elasticity. Under the condition of constant counterweight, a large force is required to separate the friction layer structure. On the contrary, if *α* is too small, the overall height *H* of the elastic skeleton increases, resulting in enhanced elasticity. Under the same counterweight, the contact between the friction layers requires a greater force. Under the condition of constant driving force, either too large or too small angles *α* of the elastic skeleton are not conducive to the contact separation of the friction layer, resulting in a decrease in the output performance of the TENG. Therefore, when the angle α of the elastic skeleton is 110°, the TENG achieves the optimal contact and separation distance of the friction layer, thereby achieving the best output performance.

In the actual marine environment, the ES-HNG is prone to tilting during operation due to the variability of ocean conditions. Therefore, an angle platform is used on the motor to simulate the effect of the tilt angle under actual wave conditions on the output performance of the TENG. By adjusting the platform to change the angle θ between the TENG and the horizontal plane, it is observed that as the angle θ gradually increases from 0° to 30°, the transferred charge, short-circuit current, and open-circuit voltage of the TENG gradually decreases, as shown in Figure S6. When angle θ is 0°, the TENG is in a state of vertical motion, allowing the weight of the magnet to fully impact the TENG and ensure comprehensive contact with the friction layers. However, as the angle θ increases gradually, the ES-HNG deviates from vertical motion, causing the weight of the magnet to not fully impact the TENG, resulting in a gradual decrease in output performance. In addition, in the actual marine environment, varying wave forces can alter the motion state and velocity of the ES-HNG. Therefore, the impact of wave force on the TENG output performance is simulated by the acceleration of the motor. First, the displacement of the linear motor is fixed at 70 mm; then, the acceleration is increased from 5 m/s^2^ to 8 m/s^2^.

The results show that the transferred charge, short-circuit current, and open-circuit voltage of the TENG increase from 1.82 μC to 2.86 μC, from 226.10 μA to 702.46 μA, and from 627.15 V to 1303.42 V (Figure S7). The interplay between the tilt angle θ and acceleration on the TENG is further explored to understand their combined effect. The output results of the transferred charge and short-circuit current are shown in Figure 2f,g. The results show that the output performance of the TENG varies under different angle and acceleration conditions. The overall trend of output performance increases as the angle θ decreases and the acceleration increases, with the optimal tilt angle θ being 0° and the optimal acceleration being 8 m/s^2^. Finally, employing optimal parameters, a series of different sizes of load resistors are used to evaluate the impedance matching and power of the TENG. The current gradually decreases as the external load resistance increases, and the output power first increases and then decreases. A peak power of 42.68 mW is obtained under a 500 kΩ load resistance, as shown in Figure 2h. The average power of 1.17 mW is obtained under a 3 MΩ load resistance, as shown in Figure S8. Even after operating for 9100 s, the TENG still showed outstanding output stability, demonstrating an extended service life and strong anti-wear performance (Figure S9).

**Figure 3:**
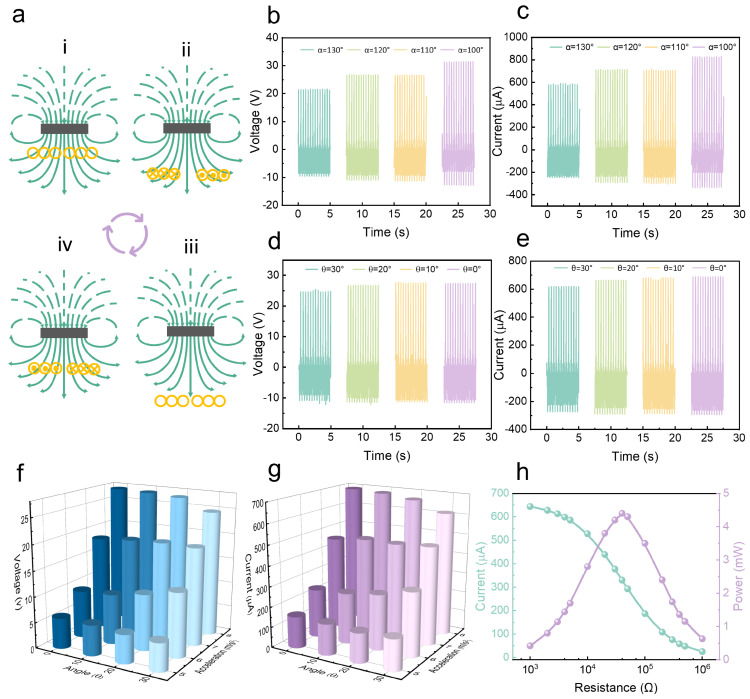
The output performance of the EMG under different parameters: (**a**) schematic diagram of the working principle of the EMG; the effect of the elastic skeletal angle α change on (**b**) output open-circuit voltage and (**c**) output short-circuit current; the effect of the tilt angle θ change on EMG (**d**) output open-circuit voltage and (**e**) output short-circuit current; the effect of acceleration and tilt angle θ on the (**f**) output open-circuit voltage and (**g**) output short-circuit current; (**h**) the output current and power-resistance change curve of the EMG.

The working principle of the EMG is shown in Figure 3a. At the initial stage, there is no change in the magnetic flux in the coil, as shown in Figure 3a(i). When the magnet ascends, the magnetic flux in the coil decreases, thereby generating a current, as shown in Figure 3a(ii). Subsequently, as the magnet continues to move upward, there is no change in the magnetic flux within the coil, resulting in no current being generated, as shown in Figure 3a(iii). When the magnet approaches the coil again, the magnetic flux in the coil increases, and a current in the opposite direction is generated in the coil, as shown in Figure 3a(iv). To investigate the factors affecting the output performance of the EMG, the same experimental platform is also used as shown in Figure 2a. First, the influence of the change in the angle α of the elastic skeleton on the output performance of the EMG is investigated. As α decreases from 130° to 100°, both the open-circuit voltage and the short-circuit current show an increasing trend. When the angle α is 100°, the open-circuit voltage and short-circuit current reach their optimal output performances, which are 31.39 V and 828.36 µA, respectively, as shown in Figure 3b,c. The output performance of the EMG complies with the following equation:

(2) $$V_{OC}=-N\frac{d\Phi }{dt},$$

(3) Φ=ΔBS,

(4) $$I_{SC}=\frac{V_{OC}}{R},$$

where Φ represents the magnetic flux inside the coil, *B* is the magnetic flux density, *S* denotes the area of the coil, *N* stands for the number of turns of the coil, and *R* is the internal resistance of the coil. This trend is primarily due to the fact that as α decreases, the overall height of the elastic skeleton increases, the movement distance between the magnet and the coil increases, the change in *B* increases, the change in Φ within the coil increases, and the open-circuit voltage and short-circuit current of the EMG increase accordingly. This is different from the trend of output performance changes in the TENG, mainly due to the differences in the working principles of the TENG and EMG. Considering that the TENG plays a key role in energy collection, to achieve the maximum performance output, an elastic skeleton with α of 110° is finally chosen as the support for the ES-HNG.

Next, the effect of the tilt angle θ of the ES-HNG on the output performance of the EMG is investigated by adjusting the angle platform. The results show that as the angle θ gradually increases, both open-circuit voltage and short-circuit current decrease, albeit to a minor extent. This phenomenon is primarily attributed to the fact that the adjustment of the tilt angle θ does not significantly change the distance between the copper coil and the magnet, resulting in minimal changes in the magnetic flux within the coil. Therefore, there is no significant change in the output performance of the EMG, as shown in Figure 3d,e. In addition, acceleration may also have a significant impact on the output performance of the EMG. Therefore, the displacement of the linear motor is also fixed at 70 mm, and the acceleration of the linear motor is increased from 5 m/s^2^ to 8 m/s^2^. Both the short-circuit current and the open-circuit voltage show an upward trend with the increase in acceleration, as shown in Figure S10. The increase in motor acceleration raises the frequency of magnet movement, thereby enhancing output performance. Subsequently, the effect of the combined effect of tilt angle θ and acceleration on the performance output of the EMG open-circuit voltage and short-circuit current is investigated, as shown in Figure 3f,g. The results show that the output performance trend of the EMG rises with the decrease in the tilt angle θ and the increase in the acceleration, and the output performance is the best when the tilt angle θ is 0° and the acceleration is 8 m/s^2^. Finally, under the optimal motor driving conditions, the impedance matching and power of the EMG are evaluated. The peak power reaches 4.40 mW under a load resistance of 40 kΩ, as shown in Figure 3h. The average power reaches 31.78 μW under the same resistance, as shown in Figure S11.

**Figure 4:**
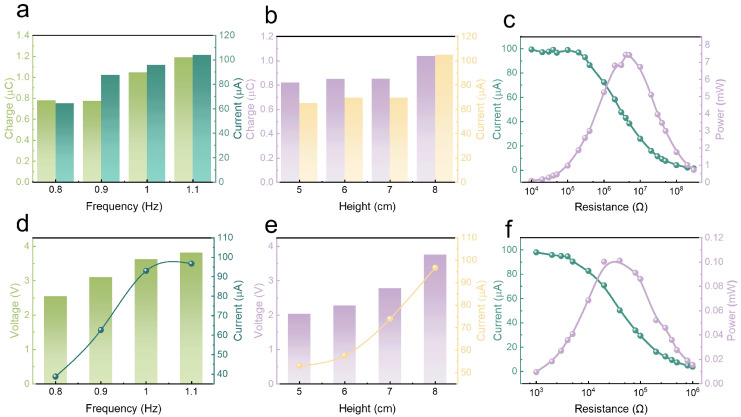
The output performance of ES-HNG under water wave conditions: the output transferred charge and short-circuit current of the TENG under different (**a**) water wave frequencies and (**b**) water wave heights; (**c**) the output short-circuit current and power-resistance change curve of the TENG under water wave conditions. The output open-circuit voltage and short-circuit current of the EMG under different (**d**) water wave frequencies and (**e**) water wave heights; (**f**) the output current and power-resistance change curve of the EMG under water wave conditions.

To evaluate the output performance of the ES-HNG under real water wave conditions, the packaged device is measured in a wave-making pool. First, the wave height is set to 8 cm, and by adjusting the parameters of the wave-making motor, the water wave frequency is gradually increased from 0.8 Hz to 1.1 Hz. With the increase in water wave frequency, the transferred charge, short-circuit current, and open-circuit voltage of the TENG all show an upward trend, increasing from 0.78 μC, 64.30 μA, and 355.68 V to 1.19 μC, 103.98 μA, and 693.60 V, respectively. The transferred charge and short-circuit current are shown in Figure 4a, and the specific waveforms of the transferred charge, short-circuit current, and open-circuit voltage changes are shown in Figure S12a–c. The acceleration in water wave frequency leads to a corresponding increase in the movement speed of the magnet, thereby augmenting the separation speed of the TENG friction layer and subsequently enhancing the output performance. Then, the frequency of the water wave is fixed at 1.1 Hz, and the height of the water wave is gradually increased. As the wave height increases from 5 cm to 8 cm, the transferred charge, short-circuit current, and open-circuit voltage of the TENG all show an upward trend, increasing from 0.82 μC, 65.28 μA, and 425.47 V to 1.04 μC, 105.10 μA, and 690.28 V, as shown in Figure 4b, and the specific waveforms are shown in Figure S12d–f. The increase in wave height means an increase in the force acting on the ES-HNG, thereby driving an increase in the movement of the magnet and further enhancing the output performance of the TENG. Finally, under the conditions of a water wave frequency of 1.1 Hz and a wave height of 8 cm, the matching impedance and power of the TENG are explored, as shown in Figure 4c. Under a load resistance of 4 MΩ, the TENG can output a peak power of up to 7.44 mW. With a load resistance of 50 MΩ, the TENG could generate an average power of 0.43 mW, as shown in Figure S13.

The output performance of the EMG in the actual water wave environment is also evaluated under the same conditions. When the wave height is fixed at 8 cm, the open-circuit voltage and short-circuit current of the EMG progressively increase with rising frequency (0.8 Hz–1.1 Hz), rising from 2.54 V and 38.82 μA to 3.82 V and 96.70 μA, as shown in Figure 4d, and the specific waveforms are shown in Figure S14a,b. Next, the frequency of the wave is set to 1.1 Hz, and the height of the wave is gradually increased from 5 cm to 8 cm. The results show that the output performance of the EMG gradually enhances, with the best outputs being open-circuit voltage reaching 3.76 V and short-circuit current reaching 96.73 μA, as shown in Figure 4e, and the specific waveforms are shown in Figure S14c,d. Then, under the most ideal water wave conditions, different specifications of load resistors are used to test the impedance matching and output power of the EMG. The test results show that under a load resistance of 40 kΩ, the EMG reaches a peak power of 0.10 mW, as shown in Figure 4f, and at the same load resistance, the average output power reaches 7.81 μW, as shown in Figure S15.

**Figure 5:**
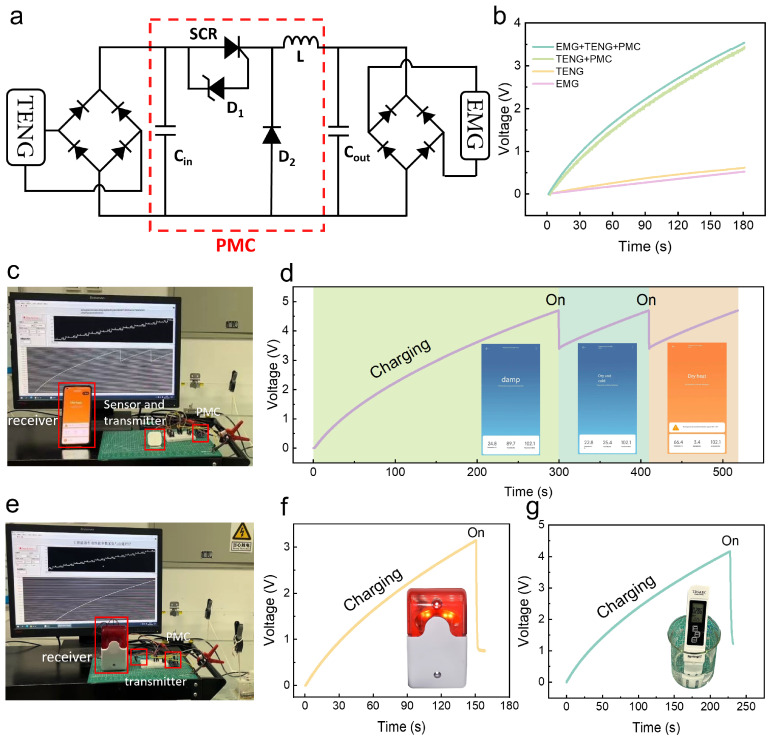
Application demonstration of the ES-HNG: (**a**) schematic diagram of the PMC principle. (**b**) The charging curve of a 1 mF capacitor under actual water wave conditions; (**c**) demonstration diagram of the ES-HNG powering the self-powered meteorological monitoring system; (**d**) voltage charging curve of the self-powered meteorological monitoring system; (**e**) demonstration diagram of the ES-HNG powering the self-powered emergency alarm system; (**f**) voltage charging curve of the self-powered alarm system; (**g**) voltage charging curve of the ES-HNG powering the water quality monitoring pen.

To achieve the maximization of energy conversion of the ES-HNG, a suitable PMC is introduced, as shown in Figure 5a. The charging curve of a 1 mF capacitor under actual water wave conditions via ES-HNG is shown in Figure 5b. Without using the PMC, the TENG can charge the capacitor voltage to 0.63 V within 180 s. However, with the PMC connected, it can charge the capacitor voltage to 3.44 V at the same time, improving the charging efficiency by 5.46 times. This demonstrates the significant role of the PMC in charging efficiency. Moreover, when the EMG charges the 1 mF capacitor independently, it achieves a voltage of 0.50 V in 180 s. Notably, when the TENG charges the 1 mF capacitor in conjunction with the EMG through the PMC, it can charge the capacitor to 3.60 V at the same time, significantly enhancing the charging effect compared to when the EMG operates independently. This result fully demonstrates the advantages of the hybrid generator in improving energy collection efficiency. To demonstrate the application of the ES-HNG in powering electronic devices, a self-powered wireless meteorological monitoring system is designed and implemented, as shown in Figure 5c. The output energy from the ES-HNG is stored in a 1 mF capacitor after going through the PMC. Subsequently, the phone and the transmitter are connected through a Wi-fi network to achieve remote transmission of meteorological signals. Following approximately 300 s of charging, the accumulated voltage in the capacitor reaches 4.70 V, enabling the temperature/humidity sensor to operate for the first time. Subsequently, at intervals of about 110 s, the phone receives a meteorological signal from the system, as shown in Video S1. The specific curve of the voltage change over time is shown in Figure 5d. Further, a self-powered wireless warning system is constructed, as shown in Figure 5e. After the ES-HNG charges a 1 mF capacitor for 150 s using the PMC, and the voltage reaches 3.13 V, the switch is closed, the transmitter is powered on and sends a signal. This triggers the remote signal receiver, making the alarm emit an auditory warning and the alarm signal light flash. The voltage change curve during this process is shown in Figure 5f and is shown in Video S2. Finally, the ES-HNG is combined with the PMC to power a water quality monitoring pen. After charging a 1 mF capacitor for about 226 s, the voltage reaches 4.13 V. At this point, closing the switch starts the water quality monitoring pen, which is shown in Video S3, and the curve of voltage changes over time is shown in Figure 5g.

## 4. Conclusions

In summary, the ES-HNG is proposed for water wave energy harvesting and marine environmental monitoring. The skeleton of the ES-HNG is manufactured using the elastic material TPU through 3D printing technology as support, which makes large-scale production of the ES-HNG possible. By combining the TENG module and the EMG module, the advantages of both can be utilized, thereby maximizing space efficiency and achieving efficient harvesting of water wave energy. After optimizing the basic parameters of the ES-HNG, the peak power generated by the ES-HNG module reaches 42.68 mW, while the peak power of the EMG module reaches 4.40 mW. In addition, the output performance of the ES-HNG is explored under different water wave conditions, with the TENG and EMG generating peak powers of 7.44 mW and 0.10 mW, respectively. Furthermore, it demonstrates that the ES-HNG powers a self-powered wireless meteorological monitoring system, a wireless alarm system, and a wireless water quality monitoring pen. This research provides a practical solution for marine wave energy harvesting and represents a promising distributed power generation technology for future marine self-powered environmental monitoring and forecasting systems.

## Data Availability

The raw data supporting the conclusions of this article will be made available by the authors on request.

## Supplementary Materials

The following supporting information can be downloaded at https://www.mdpi.com/article/10.3390/s24123770/s1: Figure S1: Physical drawing of the ES-HNG; Figure S2: Working principle diagram of the ES-HNG; Figure S3. Comparison of the output performance of ES-HNG with the previous classic works; Figure S4: The effect of different counterweight on the open-circuit voltage output performance of the TENG; Figure S5: Diagram of the relationship between the angle and height of the ES-HNG elastic skeleton; Figure S6: The impact of the tilt angle θ on the output performance of the TENG; Figure S7: The impact of the accelerations on the output performance of the TENG; Figure S8: The output current and average power–resistance change curve of the TENG; Figure S9: The durability test of the TENG under the operating time of nearly 9100 s. Figure S10: The impact of the accelerations on the output performance of the EMG; Figure S11: The output current and average power–resistance change curve of the EMG; Figure S12: The specific waveform of the TENG’s output performance under water wave conditions; Figure S13: The output current and average power–resistance change curve of the TENG in water; Figure S14: The specific waveform of the EMG’s output performance under water wave conditions; Figure S15: The output current and average power–resistance change curve of the EMG in water; Video S1: Demonstration of a self-powered temperature and humidity sensor; Video S2: Demonstration of a self-powered emergency alarm system; Video S3: Demonstration of a water quality test.
