# Supplementary material for: Elastic Self-Recovering Hybrid Nanogenerator for Water Wave Energy Harvesting and Marine Environmental Monitoring
Source: Sensors (Basel). 2024 Jun 10;24(12):3770. doi: 10.3390/s24123770 (PMC11207515; doi:10.3390/s24123770)
Supplement: Supplementary file 1 [file sensors-24-03770-s001.zip › sensors-3035493-supplementary.pdf]

sensors

Supplementary Materials for

Elastic Self-Recovering Hybrid Nanogenerator for Water Wave Energy Harvesting and Marine Environmental Monitoring

Qiuxiang Wang [1,2], Gao Yu [1,2], Ying Lou [1,2], Mengfan Li [1,2], Jiaxi Hu [1,2], Jiaodi Li [1,2], Weiqi Cui [1,2], Aifang Yu [1,2,3,*] and Junyi Zhai [1,2,3,*]

[1] Center on Nanoenergy Research, Institute of Science and Technology for Carbon Peak & Neutrality, Key Laboratory of Blue Energy and Systems Integration (Guangxi University), Education Department of Guangxi Zhuang Autonomous Region, School of Physical Science & Technology, Guangxi University, Nanning 530004, China
[2] Beijing Key Laboratory of Micro-Nano Energy and Sensor, Center for High-Entropy Energy and Systems, Beijing Institute of Nanoenergy and Nanosystems, Chinese Academy of Sciences, Beijing 101400, China
[3] School of Nanoscience and Engineering, University of Chinese Academy of Science, Beijing 100049, China
* Correspondence: yuaifang@binn.cas.cn (A.Y.); jyzhai@binn.cas.cn (J.Z.)

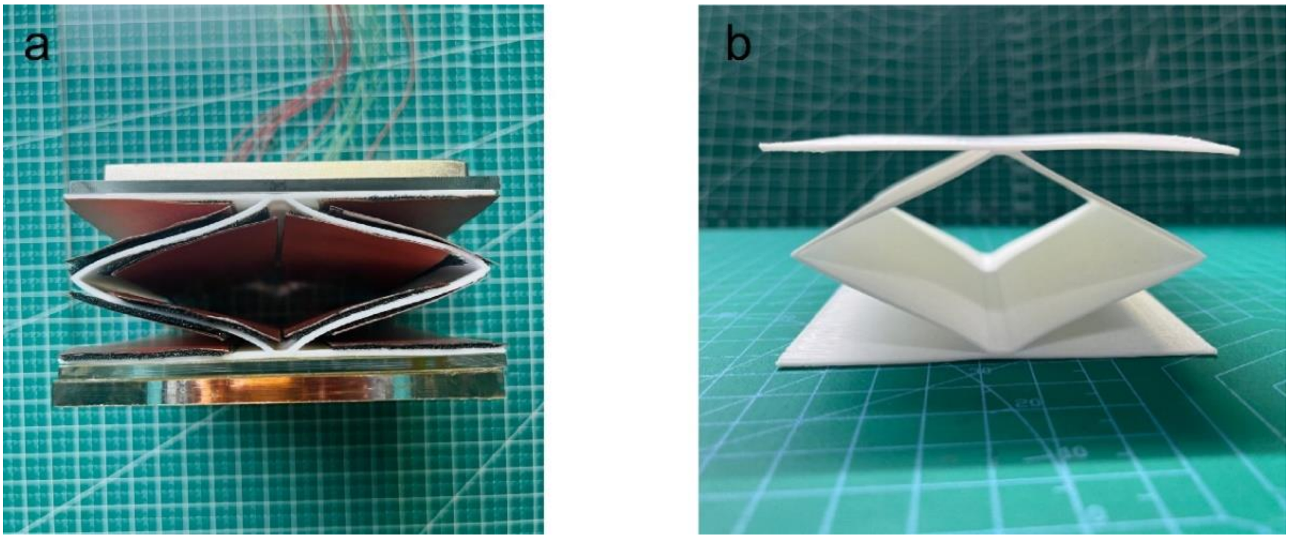

Figure S1. Physical drawing of the (**a**) ES-HNG structure and (**b**) elastic skeleton.

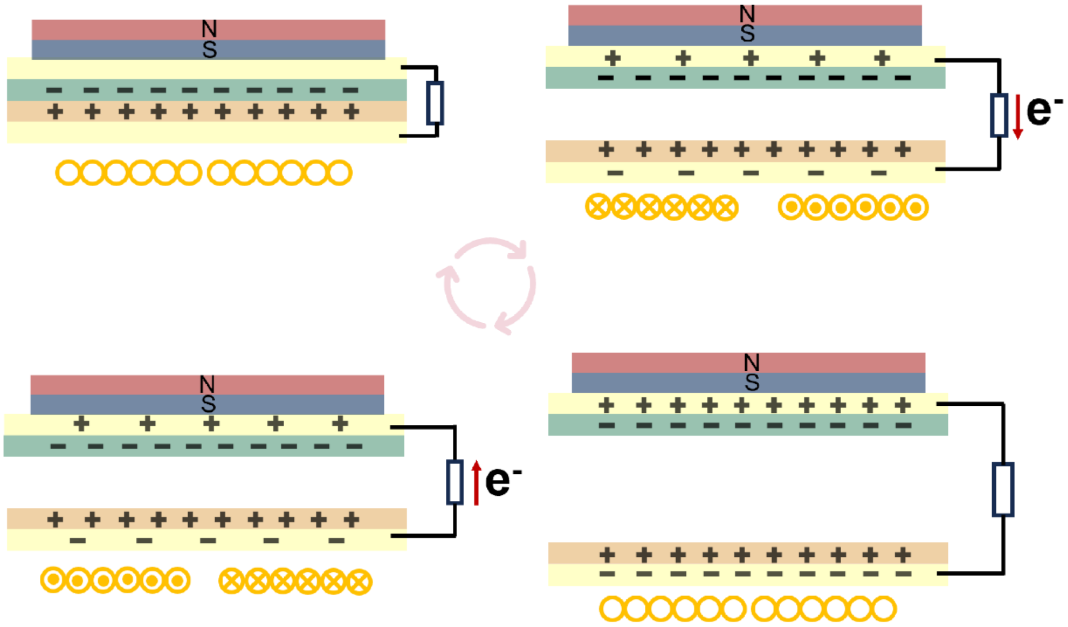

Figure S2. Working principle diagram of the ES-HNG.

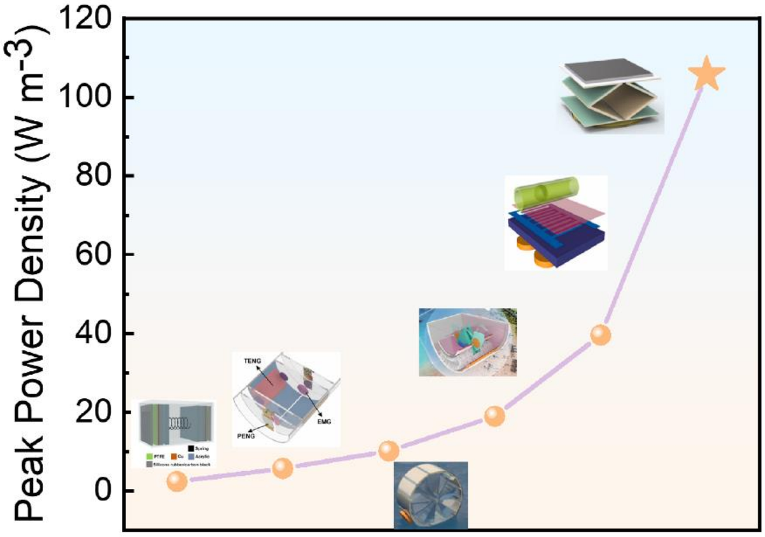

Figure S3. Comparison of the output performance of ES-HNG with the previous classic works.

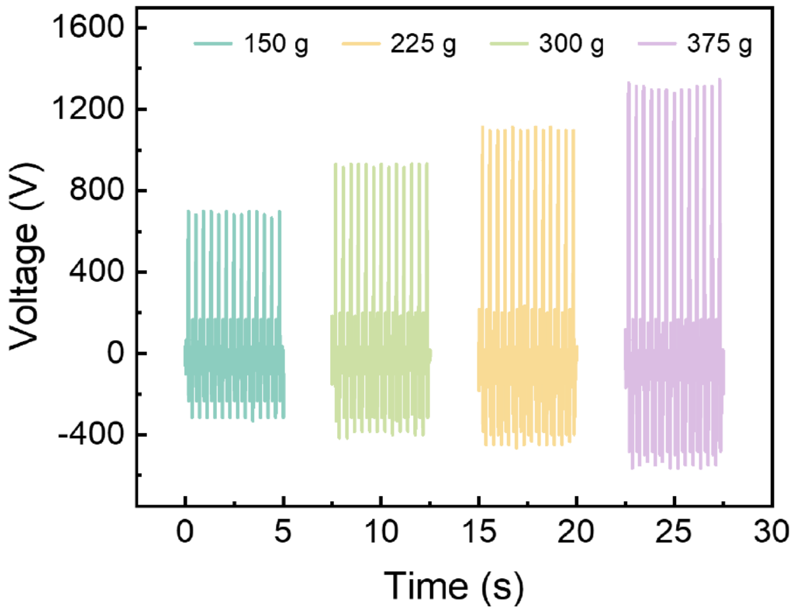

Figure S4. The effect of different counterweights on the open-circuit voltage output performance of the TENG.

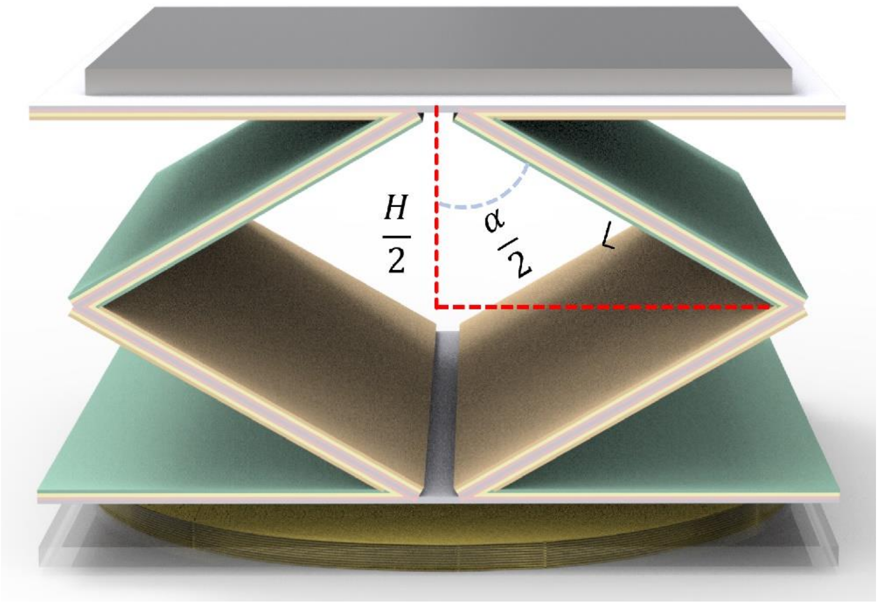

Figure S5. Diagram of the relationship between the angle and height of the ES-HNG elastic skeleton.

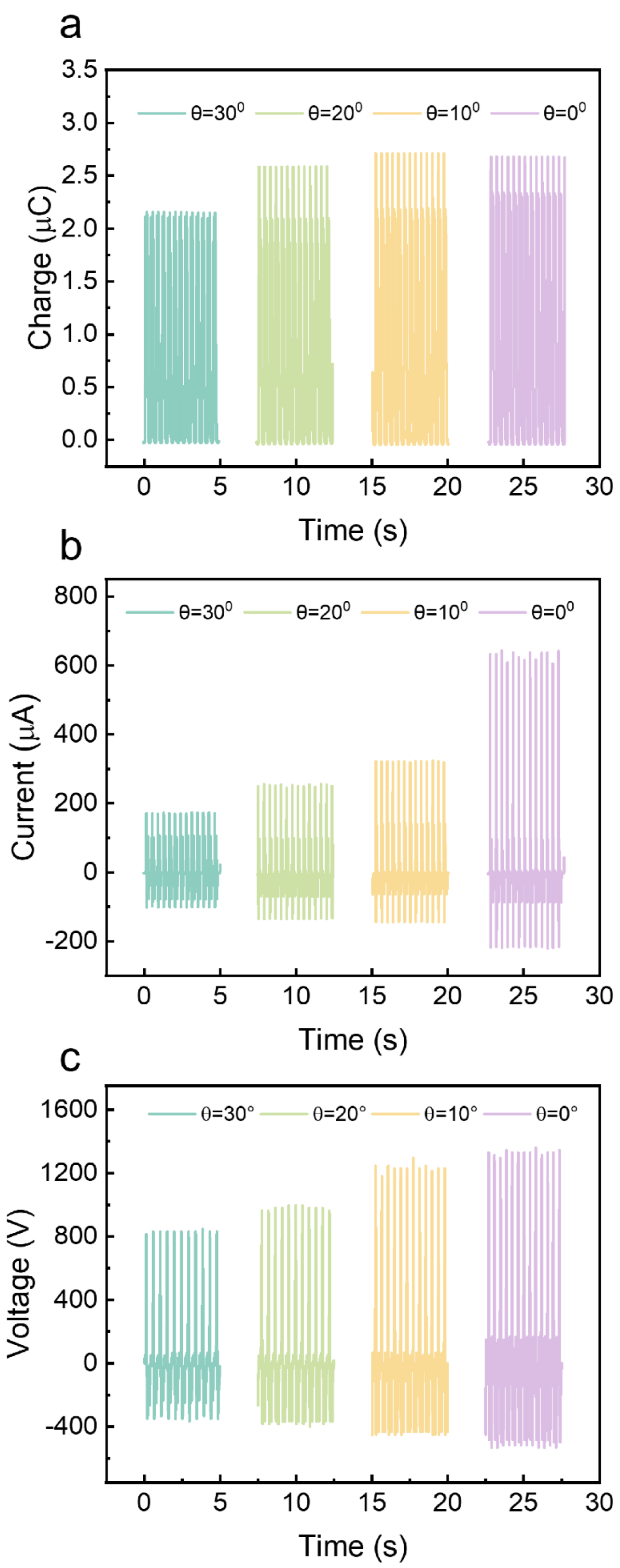

Figure S6. The impact of the tilt angle θ on the output performance of the TENG. The impact of different tilt angles θ on the (**a**) transferred charge, (**b**) short-circuit current, and (**c**) open-circuit voltage output performance.

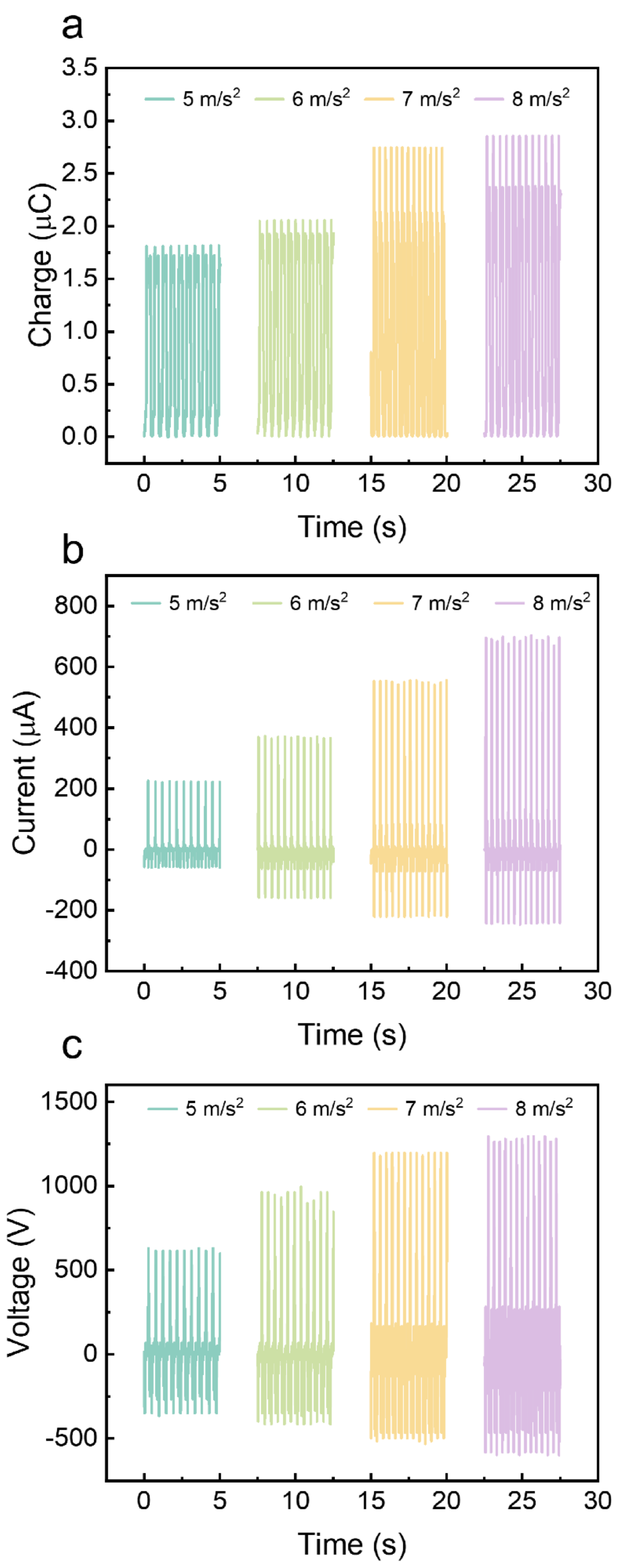

Figure S7. The impact of the accelerations on the output performance of TENG. The impact of different accelerations on the (**a**) transferred charge, (**b**) short-circuit current, and (**c**) open-circuit voltage output performance.

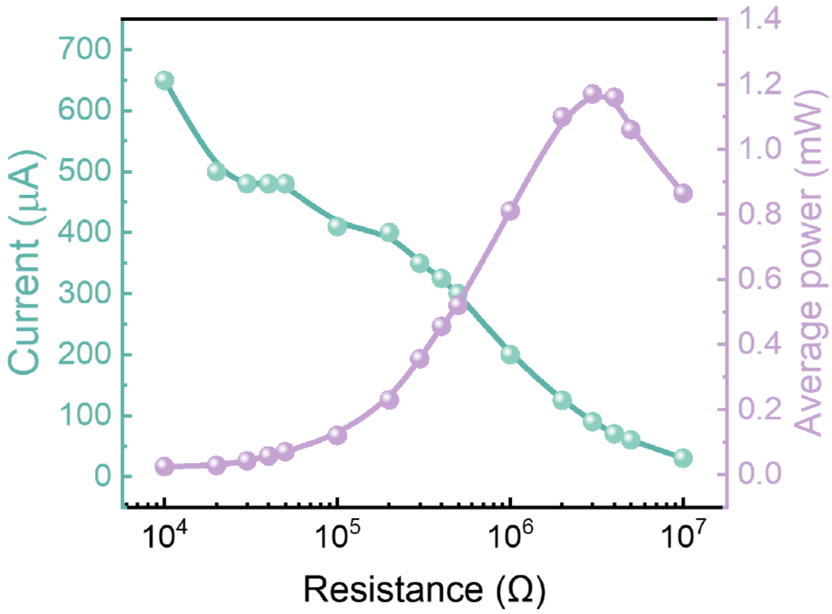

Figure S8. The output current and average power–resistance change curve of the TENG.

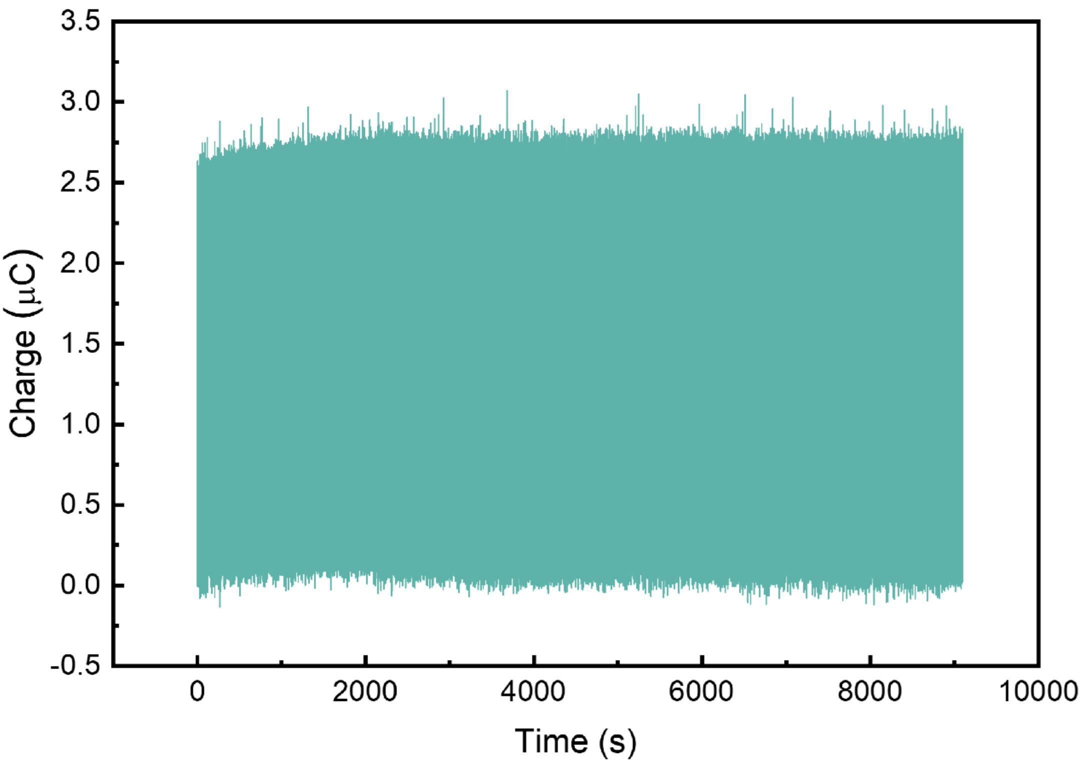

Figure S9. The durability test of TENG under the operating time of nearly 9100 s.

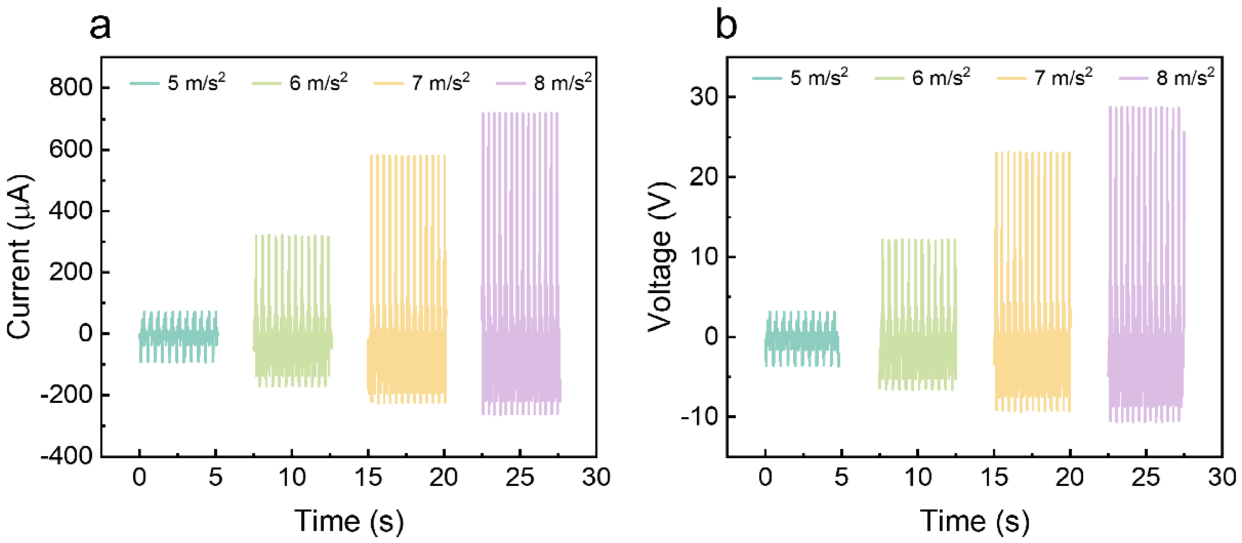

Figure S10. The impact of acceleration on the output performance of the EMG. The impact of different accelerations on the (**a**) short-circuit current and (**b**) open-circuit voltage output performance.

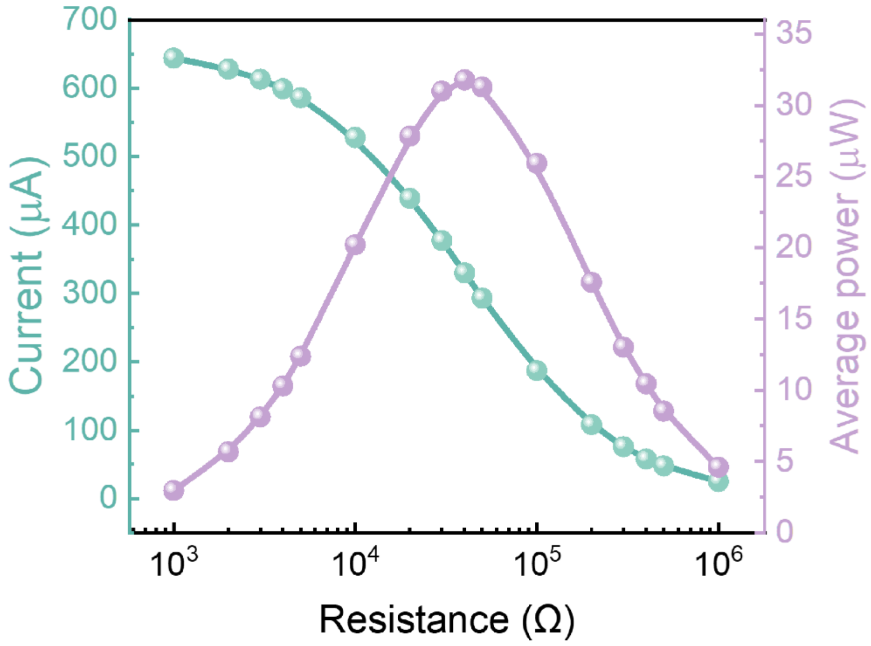

Figure S11. The output current and average power–resistance change curve of the EMG.

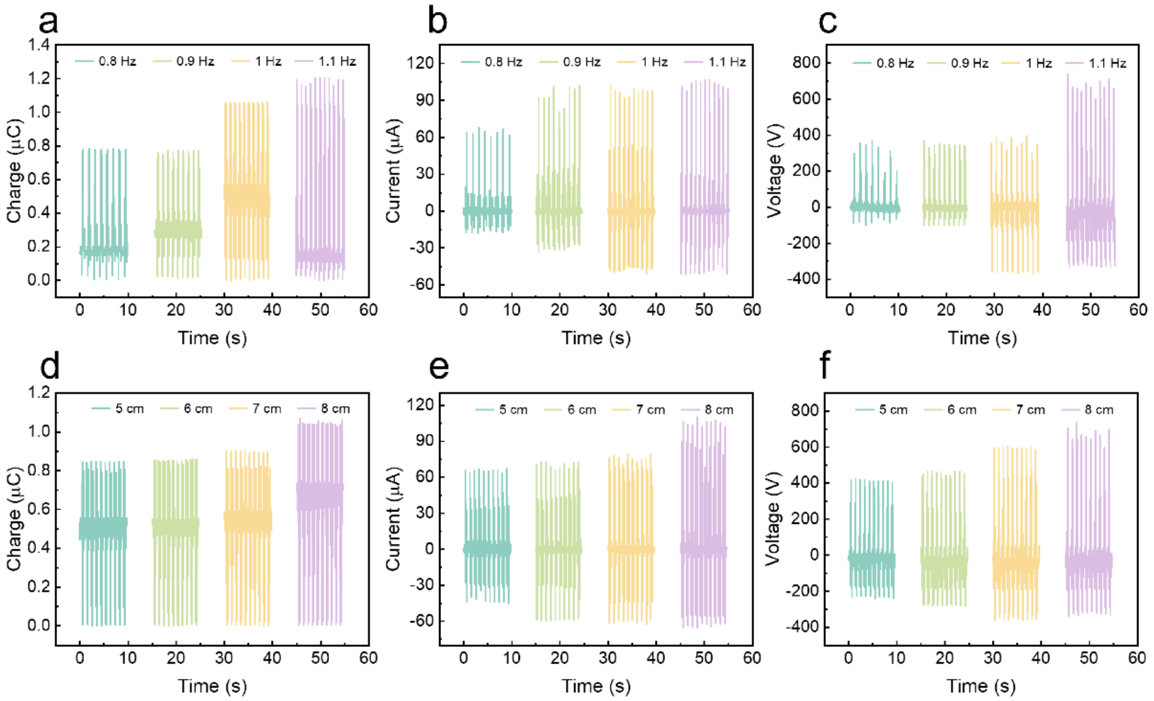

Figure S12. The specific waveform of the TENG's output performance under water wave conditions: the specific output waveforms of (**a**) transferred charge, (**b**) short-circuit current, and (**c**) open-circuit voltage under different water wave frequency conditions in TENG; the specific output waveforms of (**d**) transferred charge, (**e**) short-circuit current, and (**f**) open-circuit voltage under different water wave height conditions in TENG.

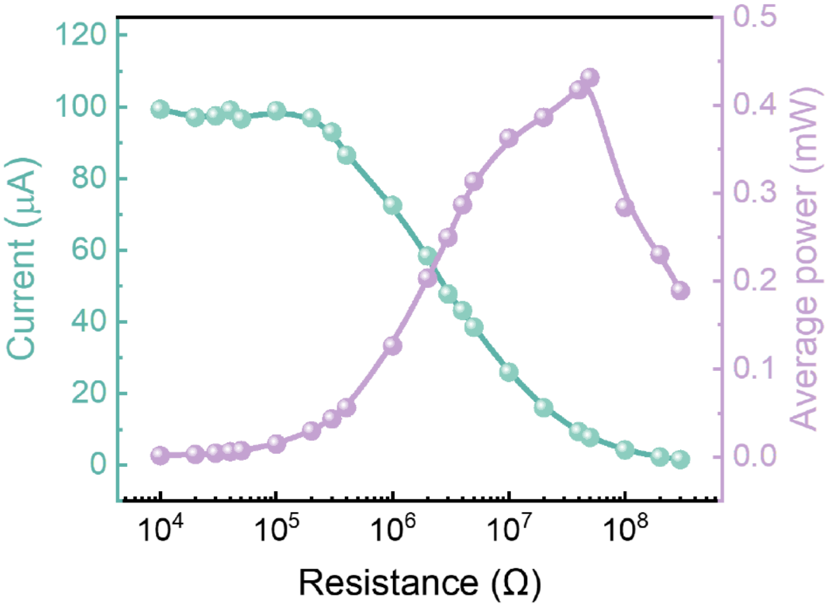

Figure S13. The output current and average power–resistance change curve of the TENG in water.

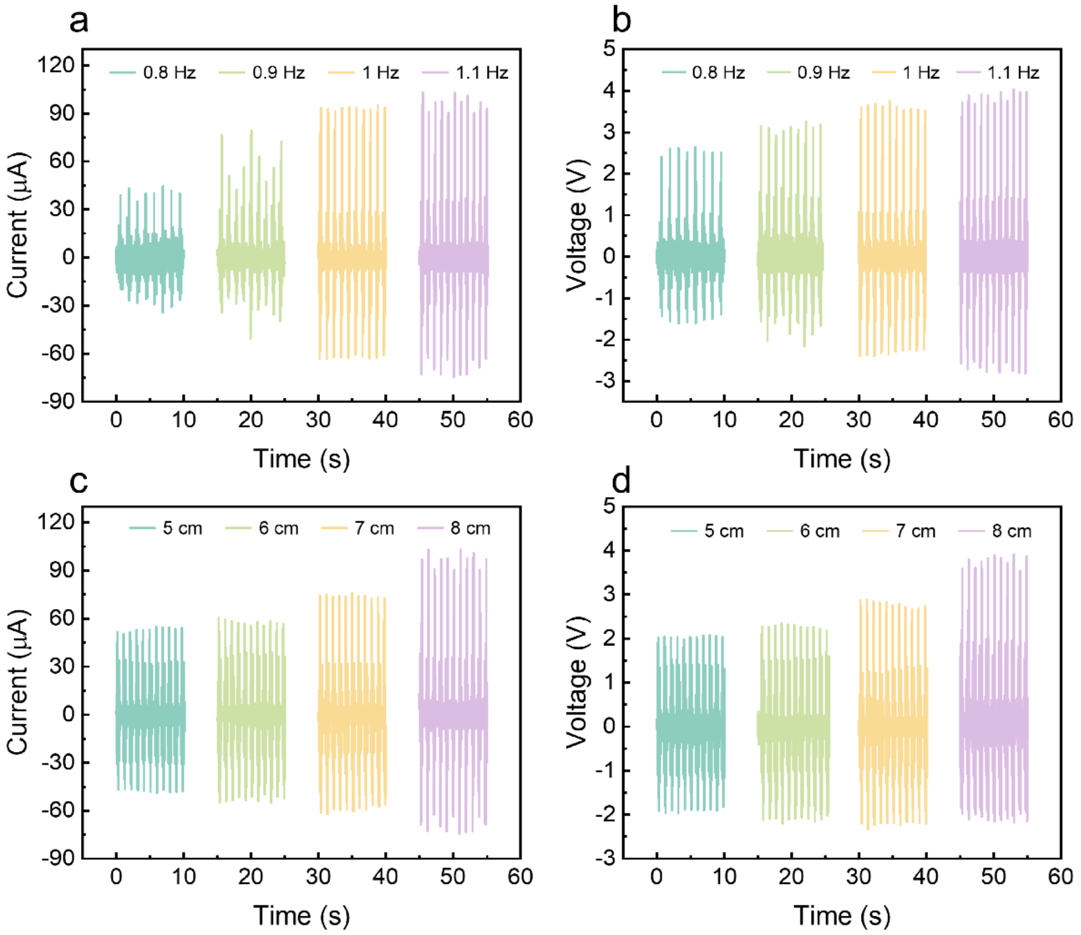

Figure S14. The specific waveform of the EMG's output performance under water wave conditions: the specific output waveforms of (**a**) short-circuit current and (**b**) open-circuit voltage under different water wave frequency conditions in EMG; the specific output waveforms of (**c**) short-circuit current and (**d**) open-circuit voltage under different water wave height conditions in EMG.

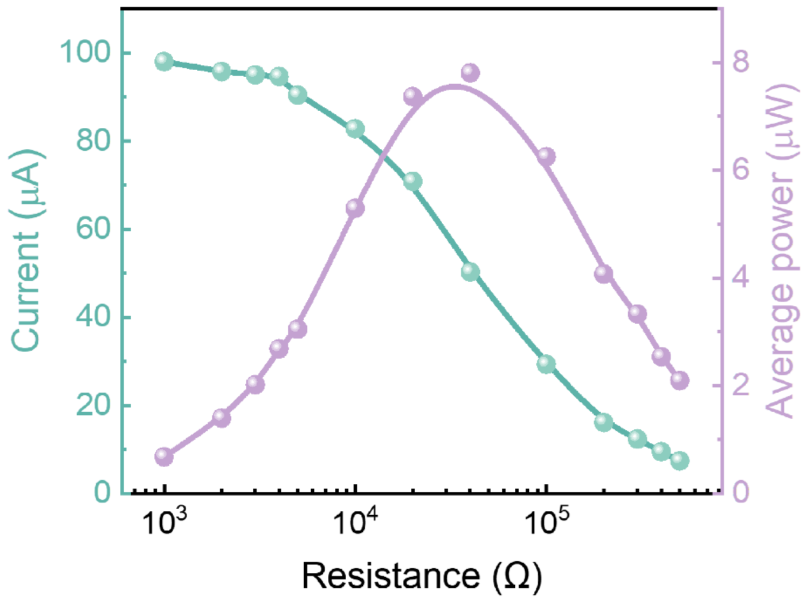

Figure S15. The output current and average power–resistance change curve of the EMG in water.

Video S1. Demonstration of a self-powered temperature and humidity sensor.

Video S2. Demonstration of a self-powered emergency alarm system.

Video S3. Demonstration of a water quality test.
